# Supplementary material for: PIP2 determines length and stability of primary cilia by balancing membrane turnovers
Source: Commun Biol. 2022 Jan 25;5:93. doi: 10.1038/s42003-022-03028-1 (PMC8789910; doi:10.1038/s42003-022-03028-1)
Supplement: Supplementary file 2 — Supplementary Material [file 42003_2022_3028_MOESM2_ESM.pdf]

Supplementary material to

PIP_2 determines length and stability of primary cilia by balancing membrane turnovers

Simon Stilling[1,2], Theodoros Kalliakoudas[3], Hannah Benninghofen-Frey[1], Takanari Inoue[2] and Björn Falkenburger[1,3,4]

1) Department of Neurology, RWTH Aachen University, Aachen, Germany

2) Department of Cell Biology, Johns Hopkins University, Baltimore, USA

3) Department of Neurology, TU Dresden, Dresden, Germany

4) JARA-Institute Molecular Neuroscience and Neuroimaging, Forschungszentrum Jülich GmbH and RWTH Aachen University, Aachen, Germany

Address correspondence to:
Prof. Björn Falkenburger
Department of Neurology
TU Dresden
Fetscherstraße 74
01307 Dresden
Germany
phone: +49 351 458 2532
email: bfalken@ukdd.de

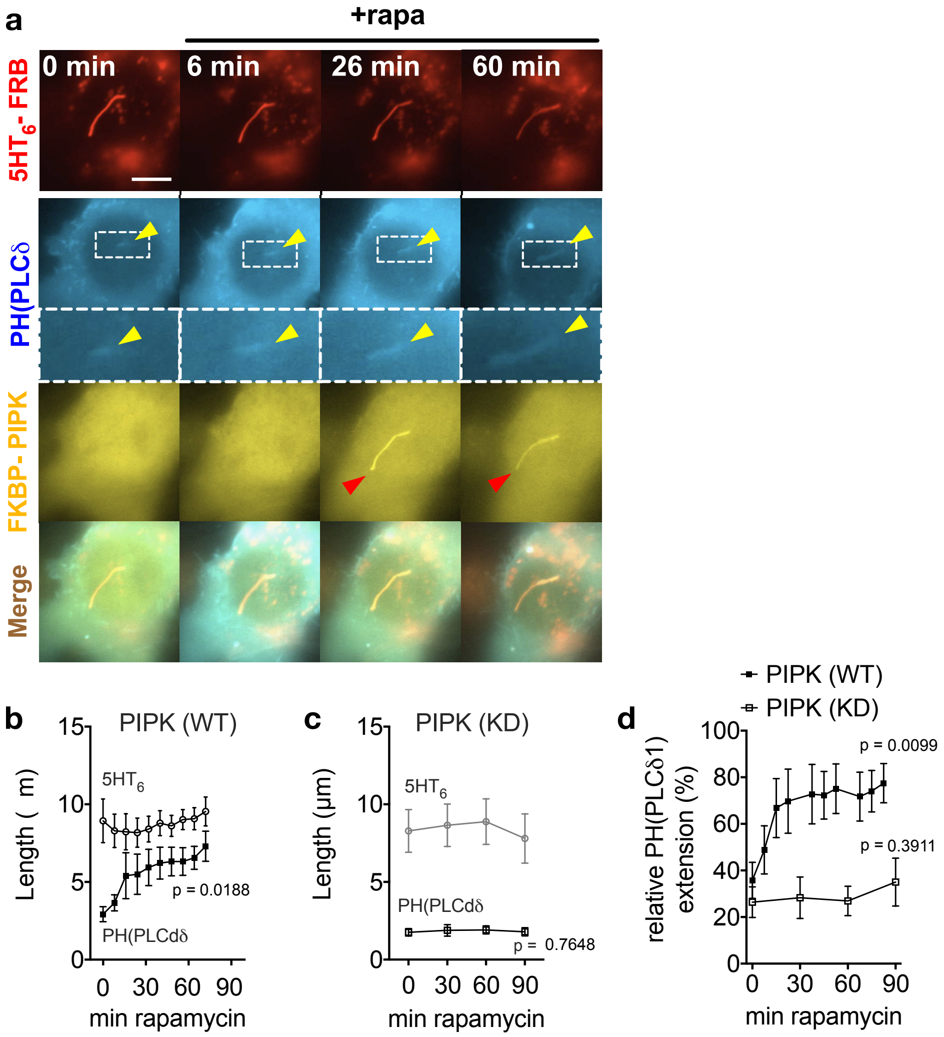

Supplement Figure S1: PIPK recruitment increases ciliary PIP_2

(a) Time-lapse images of a NIH3T3 cells expressing the ciliary marker $5HT_6$-mCherry-FRB, the PIP_2 biosensor CFP-PH(PLCδ1) and the CID tool YFP-FKBP-PIPK (WT). Images were acquired at the indicated time points after addition of 100 nM rapamycin to recruit YFP-FKBP-PIPK to $5HT_6$-mCherry-FRB. Yellow arrow heads mark ciliary PIP_2 as reported by PH(PLCδ1), and red arrowheads mark the location of the CID tool YFP-FKBP-PIPK after recruitment to the cilium. Scale bar 10 µm (b) Time course of cilium length as reported by mCherry-tagged $5HT_6$ and the length of the PH(PLCδ1) signal in n=6 cells as in (a). P-value from one-way ANOVA of PH(PLCδ1) vs. time. (c) Time course of cilium length and length of PH(PLCδ1) in n=9 cilia of cells treated as in (a) but with kinase dead (KD) PIPK instead of WT PIPK. P-value from one-way ANOVA of PH(PLCδ1) vs. time. (d) Length of PIP_2 as reported by PH(PLCδ1) expressed relative to the cilium length for the same cells as in (b) and (c), P-values from one-way ANOVA of PH(PLCδ1) vs. time.

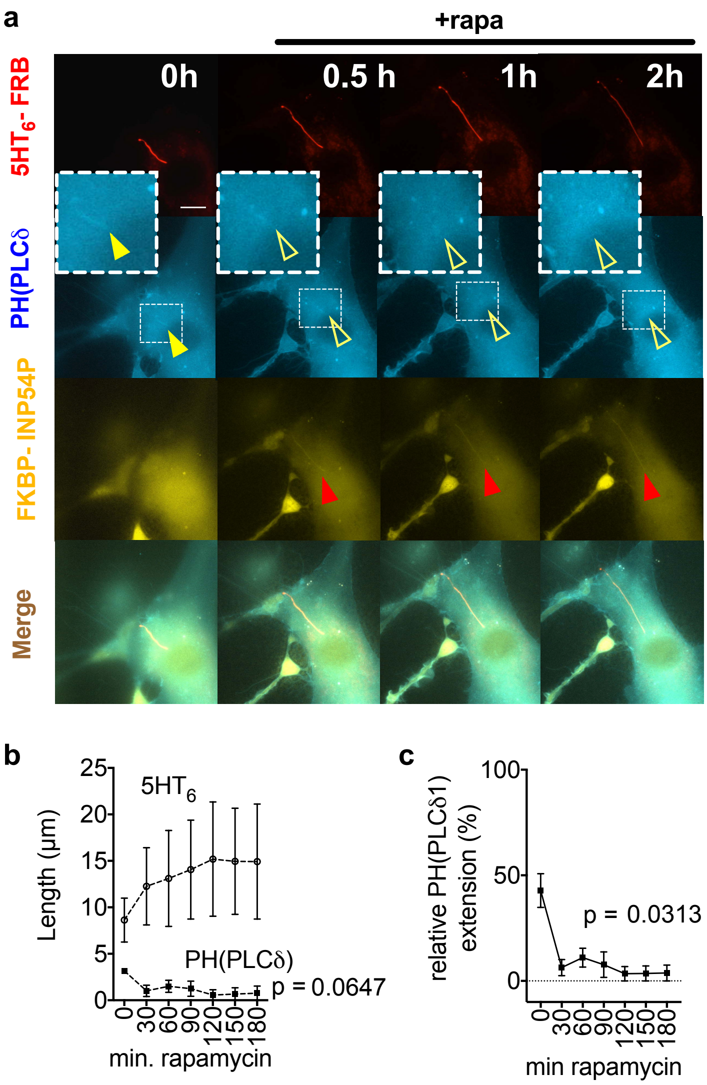

Supplementary Figure S2: Inp54p recruitment reduces ciliary PIP_2

(a) Time-lapse images of NIH3T3 cells expressing the ciliary marker $5HT_6$-mCherry-FRB, the PIP_2 biosensor CFP-PH(PLCδ1) and the CID tool YFP-FKBP-Inp54p. Images were acquired at the indicated time points after addition rapamycin (100 nM final). Closed yellow arrowheads mark the initial location of PH(PLCδ1) in the cilium; open yellow arrowheads indicate the same position as PH(PLCδ1) dissociates from the cilium. Red arrowheads mark recruitment of YFP-FKBP-Inp54p to the cilium. Scale bar 10 µm. (b) Time course of cilium length as reported by mCherry-tagged $5HT_6$ and length of PH(PLCδ1) in n=4 cells treated as in (a). P-value from one-way ANOVA of PH(PLCδ1) vs. time. (c) Length of PIP_2 as reported by PH(PLCδ1) expressed relative to the cilium length for the same cells as in (b). P-value from one-way ANOVA of PH(PLCδ1) vs. time.

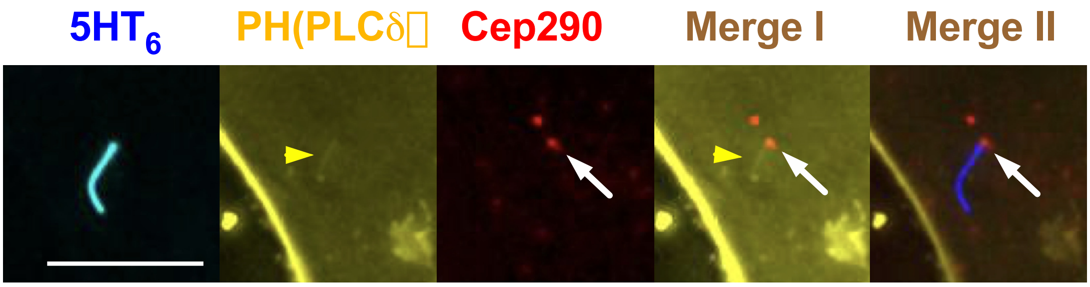

Supplementary Figure S3: PIP_2 is localised distal of CEP290

Live cell images of a NIH3T3 cell expressing the ciliary marker $5HT_6$-CFP, the PIP_2 reporter YFP-PH(PLCδ1) and mCherry-Cep290 to label the transition zone. The white arrow indicates the proximal region of the cilium marked by Cep290, the yellow arrow head indicates the location of PIP_2 in the cilium. Scale bar 10 µm.

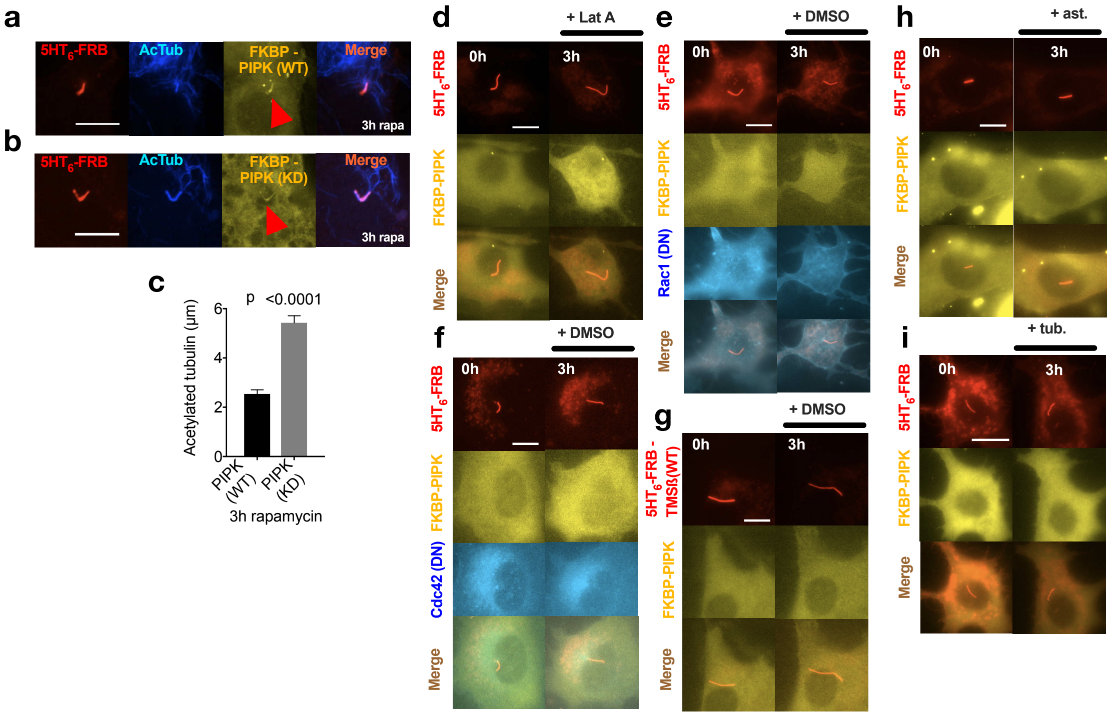

Supplementary Figure S4: Control experiments

(a-c) Validation of 5HT$_6$-mCherry by staining for acetylated tubulin. (a) Example of a NIH3T3 cell expressing the ciliary marker 5HT$_6$-mCherry-FRB and the CID tool YFP-FKBP PIPK (WT) fixed 3 h after addition rapamycin (100 nM final) and stained against acetylated tubulin (AcTub). Scale bar 10 µm. (b) Example of a cell treated as in (a) but with KD instead of WT PIPK. (c) Length of n = 53-61 cilia as defined by acetylated tubulin staining in 3 independent experiments as in (a) respectively (b), t-test.
(d-i) Images of controls for manipulations used in Figures 2 and 3 . (d) Time-lapse images of NIH3T3 cells expressing the ciliary marker 5HT$_6$-mCherry-FRB and the CID tool YFP-FKBP-PIPK acquired 0 h and 3 h after adding addition latrunculin (200 nM final) and DMSO as control instead of rapamycin. (e, f) Example images of cells as in (d), but expressing in addition CFP-tagged DN Rac1 (e) or DN Cdc42 (f), i.e. cells treated with DMSO instead of rapamycin. (g) Time-lapse images of NIH3T3 cells expressing 5HT$_6$-mCherry-FRB-TMSß for actin depolymerisation and YFP-FKBP-PIPK. Images were acquired 0 h and 3 h after addition of DMSO instead of rapamycin. (h-i) Time-lapse images of NIH3T3 cells expressing the ciliary marker 5HT$_6$-mCherry-FRB and the CID tool YFP-FKBP-PIPK acquired 0 h and 3 h after addition of DMSO as control instead of rapamycin and in addition alisertib (200 nM final, h) or tubacin (20 µM final, i). All scale bars 10 µm.

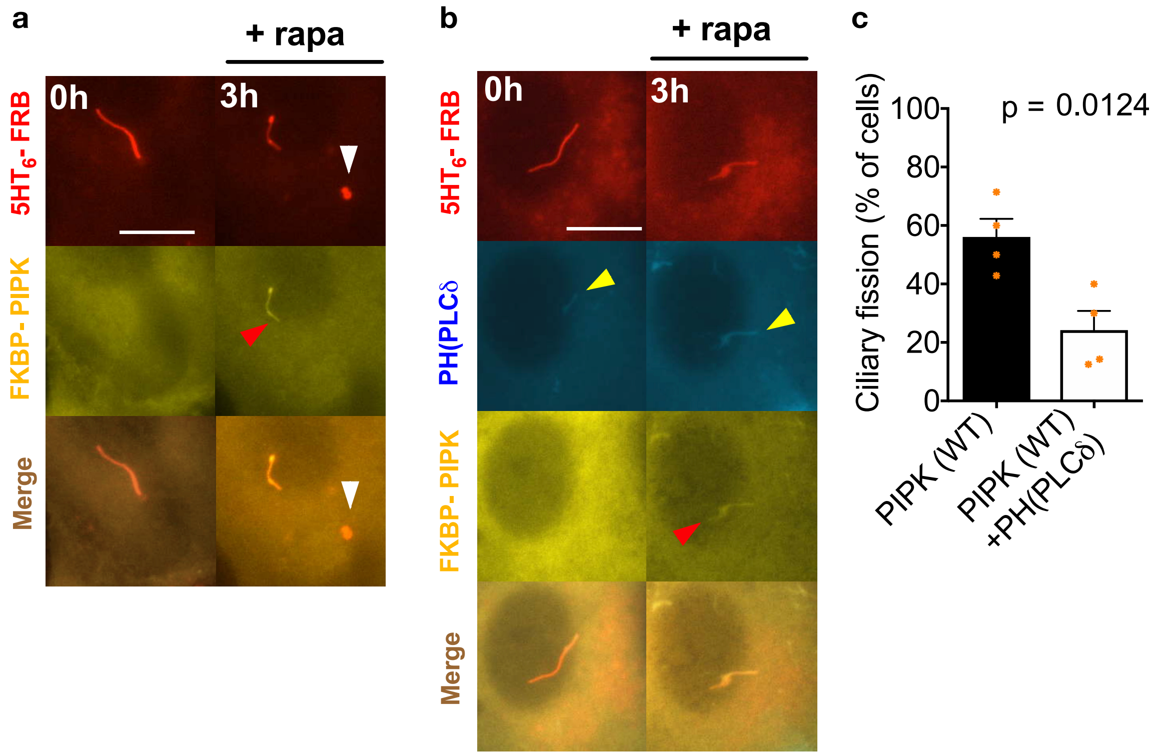

Supplementary Figure S5: Sequestering PIP_2 inhibits ciliary fission

(a, b) Time-lapse images of NIH3T3 cells expressing the ciliary marker $5HT_6$-mCherry-FRB, the CID tool YFP-FKBP-PIPK (WT), and in (b) the PIP_2 biosensor CFP-PH(PLCδ1). Images were acquired after 0 and 3 h with rapamycin (100 nM final). White arrowheads mark ciliary vesicles, yellow arrowheads mark ciliary PIP_2 as reported by PH(PLCδ1), red arrowheads mark recruitment of PIPK. Scale bars 10 µm. (c) Quantification of ciliary fission in 32-35 cilia from n = 4 independent experiments, t-test.

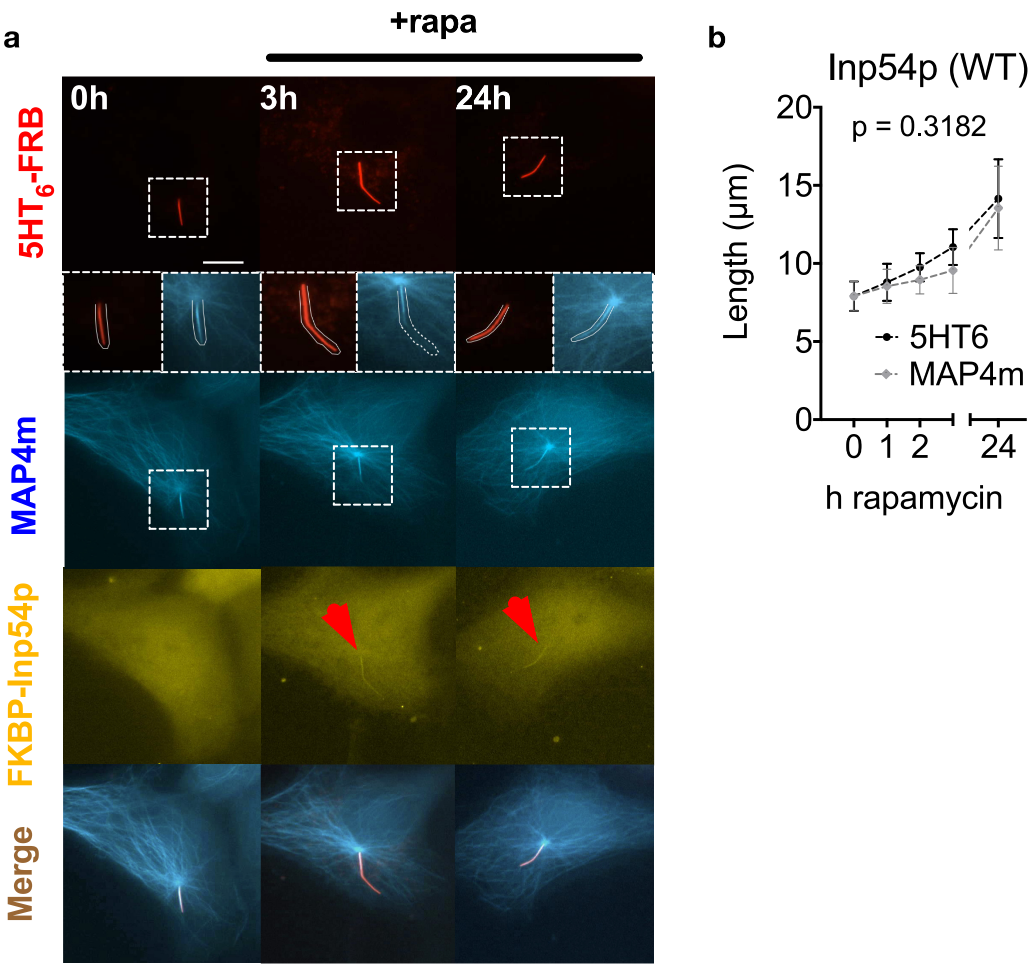

Supplementary Figure S6: PIP_2 depletion leads to elongation of ciliary microtubules

(a) Time-lapse images of a NIH3T3 cell expressing the ciliary marker $5HT_6$-mCherry-FRB, the CID tool YFP-FKBP-Inp54p (WT) and the microtubule marker CFP-MAP4m. Red arrowheads mark recruitment of the CID tool to the cilium. Scale bar 10 µm. Insets are the boxed areas of the $5HT_6$-mCherry and CFP-MAP4m channels. A region was drawn around the area of the cilium as defined by $5HT_6$-mCherry. Note that CFP-MAP4m does not cover the entire cilium in the 3 h image. (b) Length of the ciliary membrane as reported by mCherry-tagged $5HT_6$ and of the ciliary microtubules as reported by MAP4m in n = 8 cilia transfected as in (a). 2-way ANOVA showed no significant interaction between the factors time and $5HT_6$ vs. MAP4m.

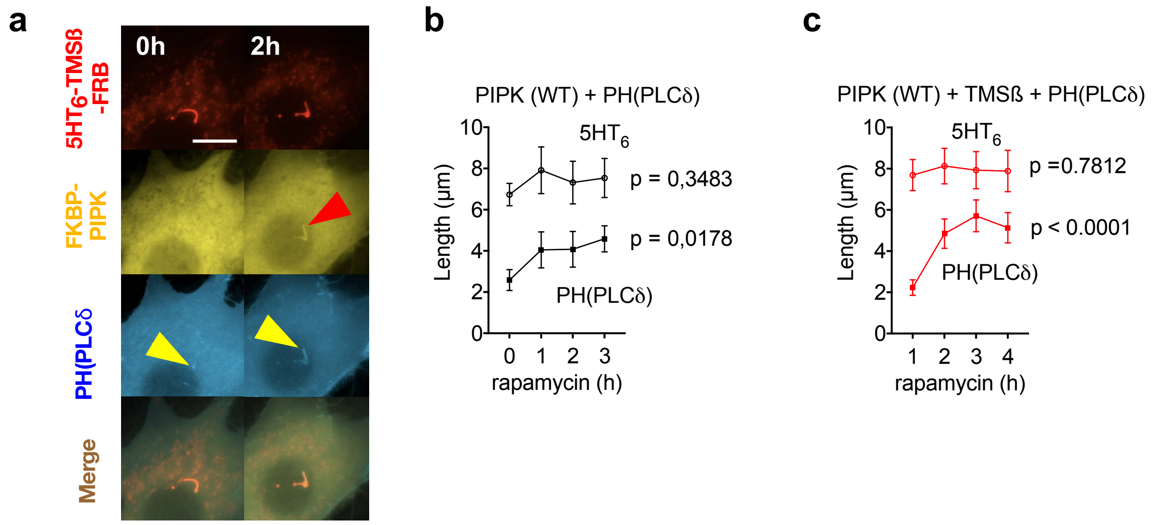

Supplementary Figure S7: TMSß does not impair PIP2 synthesis

(a) Time-lapse images of NIH3T3 cells expressing the ciliary marker $5HT_6$-mCherry-FRB-TMSß for actin depolymerisation and in addition the PIP_2 biosensor CFP-PH(PLCδ1). Red arrowhead marks recruitment of PIPK, yellow arrow heads mark ciliary PIP_2 as reported by PH(PLCδ1). Scale bars 10µm. (b) Length of n=14 cilia and length of PH(PLCδ1) in 3 independent experiments as in (a) but without expressing TMSß, one-way ANOVA for cilia length and one-way ANOVA for PH(PLCδ1). (c) Length of n=21 cilia and length of PH(PLCδ1) in 3 independent experiments as in (a), one-way ANOVA for cilia length and one-way ANOVA for PH(PLCδ1).

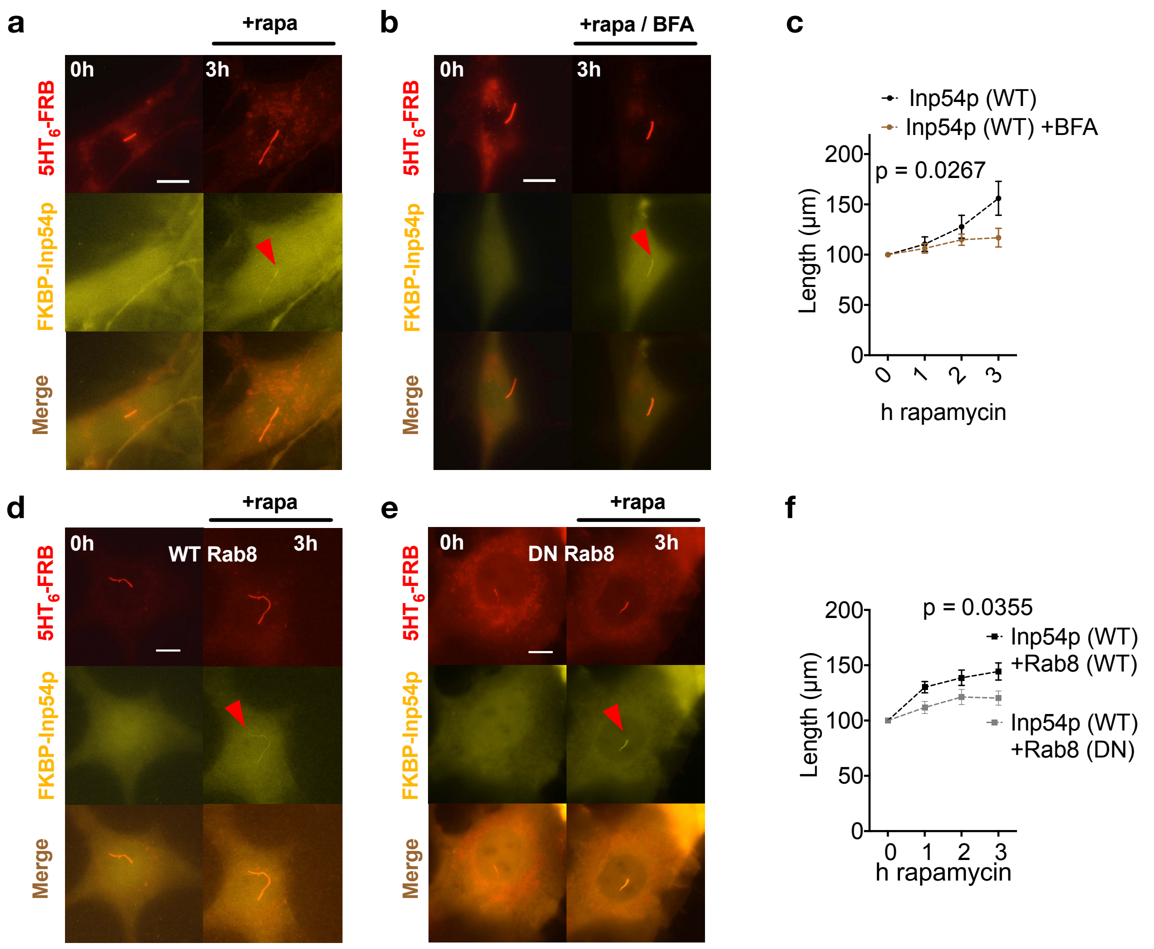

Supplementary Figure S8: Ciliary elongation requires Golgi-derived vesicles

(a, b) Time-lapse images of NIH3T3 cells expressing the ciliary marker $5HT_6$-mCherry-FRB and the CID tool YFP-FKBP-Inp54p (WT). Images were acquired 0 h and 3 h after addition of rapamycin (100 nM final) (a and b) and 100 ng/ml brefeldin A (BFA, in b) or control (in a). Red arrowheads mark recruitment of YFP-FKBP-Inp54p. (c) Time course of cilium length normalised to the length at t=0 h reported by mCherry-tagged $5HT_6$ in n=34 or n=30 cilia from 4 independent experiments as in (a) and (b), two-way ANOVA. (d, e) Time-lapse images of NIH3T3 cells expressing the ciliary marker $5HT_6$-mCherry-FRB, the CID tool YFP-FKBP-Inp54p (WT) and in addition WT Rab8 (without fluorescent label, d) or DN Rab8 (without fluorescent label, e). Red arrowheads mark recruitment of Inp54p. (f) Time course of cilium length normalised to the length at t=0 h in n=51 cilia from 4 independent experiments as in (d) or in n=48 cilia from 4 independent experiments as in (e), two-way ANOVA.

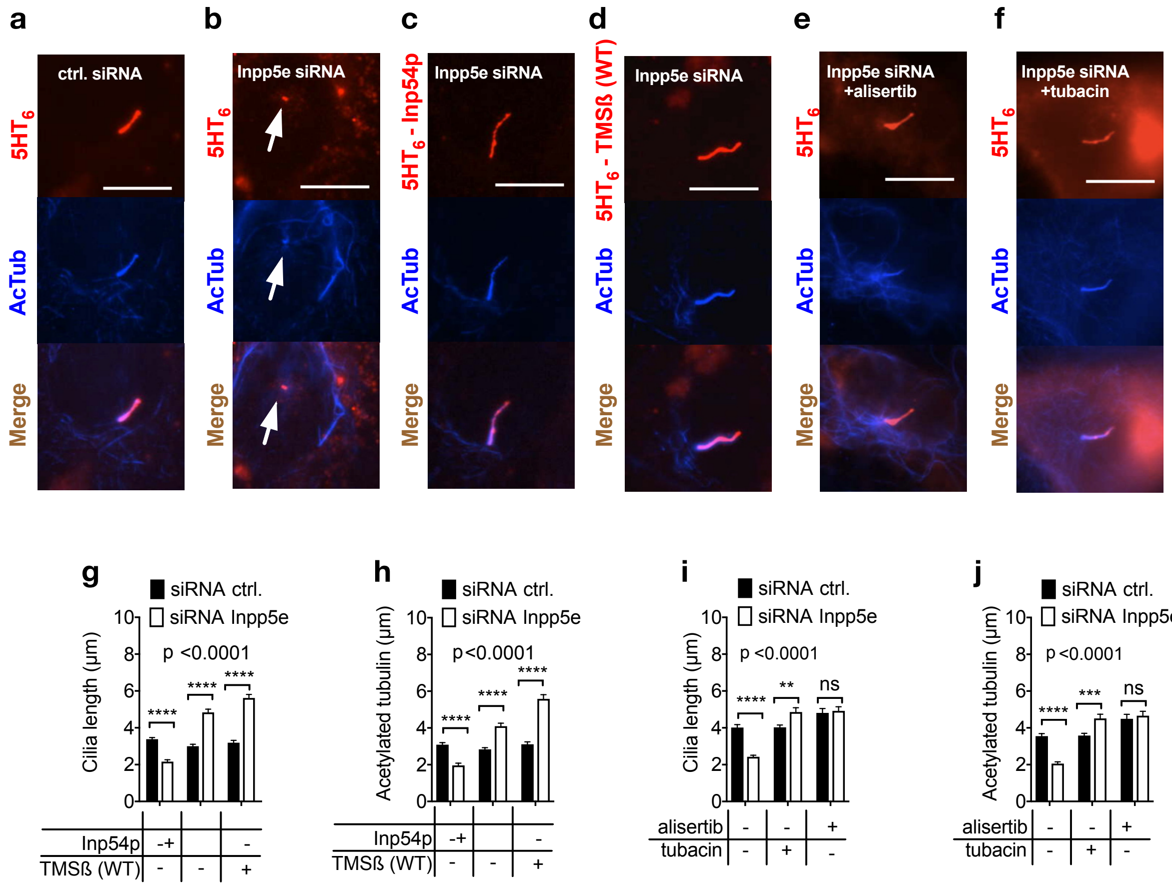

Supplementary Figure S9: Inpp5e deficiency phenotype requires PIP_2, actin polymerisation, AurkA and HDAC6

(a, b) Examples of NIH3T3 cells expressing the ciliary marker $5HT_6$-mCherry, transfected with control non-targeting siRNA (a) or siRNA for Inpp5e (b), fixed and stained against acetylated tubulin (AcTub). Scale bar 10 µm. White arrow indicates cilium. (c, d) Examples of NIH3T3 cells transfected with siRNA for Inpp5e and expressing the ciliary marker $5HT_6$-mCherry-Inp54p (c) to deplete ciliary PIP_2 or $5HT_6$-mCherry-TMSß (d) to depolymerise ciliary actin. (e-f) Examples of NIH3T3 cells transfected with siRNA for Inpp5e, expressing the ciliary marker $5HT_6$-mCherry, and incubated with alisertib (e, 200 nM final) respectively tubacin (f, 2 µM final) for 24 h. (g-h) Length of the ciliary membrane as reported by mCherry-tagged $5HT_6$ in n=99-130 cilia from 3 independent experiments (g) or by acetylated tubulin in n=60-91 cilia from 3 independent experiments (h) as in (a-d). P values represent the interaction between the factors siRNA (Ctrl. vs Inpp5e) and 5HT6 construct (Ctrl. vs. Inp54p vs. TMSß) from two-way ANOVA. The results of posthoc tests are depicted by asterisks. (i-j) Length of the ciliary membrane as reported by mCherry-tagged $5HT_6$ in n=57-84 cilia from 3 independent experiments (i) or by acetylated tubulin in n=57-83 cilia from 3 independent experiments (j) as in a, b, e and f. P values represent the two-way ANOVA interaction between the factors siRNA (Ctrl. vs Inpp5e) and treatment (Ctrl. vs. alisertib vs. tubacin). The results of posthoc tests are depicted by asterisks.
