# Supplementary material for: PIP2 determines length and stability of primary cilia by balancing membrane turnovers
Source: Commun Biol. 2022 Jan 25;5:93. doi: 10.1038/s42003-022-03028-1 (PMC8789910; doi:10.1038/s42003-022-03028-1)
Supplement: Supplementary file 3 — Description of Additional Supplementary File [file 42003_2022_3028_MOESM3_ESM.pdf]

Description of Additional Supplementary Files

File name: Supplementary Data 1
Description: Raw Data for Figures 1-6.
