# Supplementary material for: PIP2 determines length and stability of primary cilia by balancing membrane turnovers
Source: Commun Biol. 2022 Jan 25;5:93. doi: 10.1038/s42003-022-03028-1 (PMC8789910; doi:10.1038/s42003-022-03028-1)
Supplement: Supplementary file 5 — Reporting Summary [file 42003_2022_3028_MOESM5_ESM.pdf]

nature research

Corresponding author(s): Bjoern Falkenburger

Last updated by author(s): 09/12/2021

Reporting Summary

Nature Research wishes to improve the reproducibility of the work that we publish. This form provides structure for consistency and transparency in reporting. For further information on Nature Research policies, see Authors & Referees and the Editorial Policy Checklist.

Statistics

For all statistical analyses, confirm that the following items are present in the figure legend, table legend, main text, or Methods section.

n/a	Confirmed	
☐	☒	The exact sample size (*n*) for each experimental group/condition, given as a discrete number and unit of measurement
☐	☒	A statement on whether measurements were taken from distinct samples or whether the same sample was measured repeatedly
☐	☒	The statistical test(s) used AND whether they are one- or two-sided<br>*Only common tests should be described solely by name; describe more complex techniques in the Methods section.*
☐	☒	A description of all covariates tested
☐	☒	A description of any assumptions or corrections, such as tests of normality and adjustment for multiple comparisons
☐	☒	A full description of the statistical parameters including central tendency (e.g. means) or other basic estimates (e.g. regression coefficient) AND variation (e.g. standard deviation) or associated estimates of uncertainty (e.g. confidence intervals)
☐	☒	For null hypothesis testing, the test statistic (e.g. *F*, *t*, *r*) with confidence intervals, effect sizes, degrees of freedom and *P* value noted<br>*Give P values as exact values whenever suitable.*
☒	☐	For Bayesian analysis, information on the choice of priors and Markov chain Monte Carlo settings
☒	☐	For hierarchical and complex designs, identification of the appropriate level for tests and full reporting of outcomes
☒	☐	Estimates of effect sizes (e.g. Cohen's *d*, Pearson's *r*), indicating how they were calculated

Our web collection on statistics for biologists contains articles on many of the points above.

Software and code

Policy information about availability of computer code

Data collection	Olympus imaging software xcellence (2.0)
Data analysis	ImageJ (NIH, 1.52) and GraphPad Prism 6 (GraphPad Software, La Jolla, USA)

For manuscripts utilizing custom algorithms or software that are central to the research but not yet described in published literature, software must be made available to editors/reviewers. We strongly encourage code deposition in a community repository (e.g. GitHub). See the Nature Research guidelines for submitting code & software for further information.

Data

Policy information about availability of data

All manuscripts must include a data availability statement. This statement should provide the following information, where applicable:

- Accession codes, unique identifiers, or web links for publicly available datasets
- A list of figures that have associated raw data
- A description of any restrictions on data availability

All data is included in the manuscript. Raw data are available from the corresponding author upon reasonable request.

Field-specific reporting

Please select the one below that is the best fit for your research. If you are not sure, read the appropriate sections before making your selection.

☒ Life sciences ☐ Behavioural & social sciences ☐ Ecological, evolutionary & environmental sciences

For a reference copy of the document with all sections, see nature.com/documents/nr-reporting-summary-flat.pdf

Life sciences study design

All studies must disclose on these points even when the disclosure is negative.

Sample size	Sample sizes were determined by previous experience in this type of research and not by an explicit sample size calculation.
Data exclusions	No data were exluded.
Replication	Experiments were replicated at least three times.
Randomization	No randomisation necessary.
Blinding	Investigators were blinded during image analalysis but not during cell culture and image acquisition.

Reporting for specific materials, systems and methods

We require information from authors about some types of materials, experimental systems and methods used in many studies. Here, indicate whether each material, system or method listed is relevant to your study. If you are not sure if a list item applies to your research, read the appropriate section before selecting a response.

Materials & experimental systems

n/a	Involved in the study	
☐	☒	Antibodies
☐	☒	Eukaryotic cell lines
☒	☐	Palaeontology
☒	☐	Animals and other organisms
☒	☐	Human research participants
☒	☐	Clinical data

Methods

n/a	Involved in the study	
☒	☐	ChIP-seq
☒	☐	Flow cytometry
☒	☐	MRI-based neuroimaging

Antibodies

Antibodies used	monoclonal primary antibody against acetylated tubulin (Sigma-Aldrich, Cat# T6793) and a DyLight 405-labelled goat anti-mouse IgG (ThermoFisher, Cat# 35501BID) secondary antibody
Validation	The antibody was used by numerous other groups, including a laboratory in the same institution. It was also used in publications on the subject.

Eukaryotic cell lines

Policy information about cell lines

Cell line source(s)	Institute of Biochemistry, RWTH Aachen University
Authentication	validated by IDEXX GmbH, Ludwigsburg, Germany using STR profiling in January 2018
Mycoplasma contamination	cell lines are tested for mycoplasma twice yearly or if suspected.
Commonly misidentified lines (See ICLAC register)	NIH3T3 cells were used because they are frequently used for studies on cilia and because it is known that they form cilia reliably.
